# Release of a Poorly Soluble Drug from Hydrophobically Modified Poly (Acrylic Acid) in Simulated Intestinal Fluids

**DOI:** 10.1371/journal.pone.0140709

**Published:** 2015-10-16

**Authors:** Patrik Knöös, Anna V. Svensson, Stefan Ulvenlund, Marie Wahlgren

**Affiliations:** 1 Department of Chemistry, Division of Physical Chemistry, Lund University, Lund, Sweden; 2 Teknologisk Institut, Taastrup, Denmark; 3 CR Competence AB, Lund, Sweden; 4 Department of food technology engineering and nutrition, Lund University, Lund, Sweden; Aristotle University of Thessaloniki, GREECE

## Abstract

A large part of new pharmaceutical substances are characterized by a poor solubility and high hydrophobicity, which might lead to a difference in drug adsorption between fasted and fed patients. We have previously evaluated the release of hydrophobic drugs from tablets based on Pemulen TR2 and showed that the release can be manipulated by adding surfactants. Here we further evaluate the possibility to use Pemulen TR2 in controlled release tablet formulations containing a poorly soluble substance, griseofulvin. The release is evaluated in simulated intestinal media that model the fasted state (FaSSIF medium) or fed state (FeSSIF). The rheology of polymer gels is studied in separate experiments, in order to gain more information on possible interactions. The release of griseofulvin in tablets without surfactant varied greatly and the slowest release were observed in FeSSIF. Addition of SDS to the tablets eliminated the differences and all tablets showed a slow linear release, which is of obvious relevance for robust drug delivery. Comparing the data from the release studies and the rheology experiment showed that the effects on the release from the different media could to a large extent be rationalised as a consequence of the interactions between the polymer and the surfactants in the media. The study shows that Pemulen TR2 is a candidate for controlled release formulations in which addition of surfactant provides a way to eliminate food effects on the release profile. However, the formulation used needs to be designed to give a faster release rate than the tablets currently investigated.

## Introduction

A majority of new active substances developed by the pharmaceutical industry are characterized by a high hydrophobicity and a low water solubility [1, 2]. As a result, these often show a low bioavailability and there is a need to develop new formulation strategies [2, 3]. Moreover, these substances often show a large variability in uptake from the intestine due to the varying composition of the intestinal fluid between fasted or fed states [4–6]. We have previously studied the possibility to use commercially available hydrophobically modified poly (acrylic acid) (HMPAA) in tablet formulations for controlled release [7–9]. The studies have focussed on Pemulen TR 2. This polymer consists of poly (acrylic acid) cross-linked with allylpentaerytritol and modified with grafted C10-C30 alkyl-chains. The polymer has shown promising results with hydrophobic drugs where an almost ideal zero order release rate was achieved until the tablets were completely dissolved [7, 9]. However, in other cases Pemulen has shown a tendency to give to slow release but by mixing Pemulen and Carbopol a pharmaceutical relevant profile was obtained [10]

The dissolution of swelling polymer tablets has been studied extensively before and involves solvent penetration, swelling and disentanglement of the polymer chains [11–13]. The formation and thickness of the concentrated to semi-dilute viscous polymer solution surrounding the dry tablet core, the so-called "gel layer", is considered to determine the dissolution rate [12] and is dependent on the mechanical strength of the gel layer and the applied convection [14–17]. This implies that changes in external factors, e.g. pH and/or amphiphilic content, that affect the gel layer properties could potentially affect the dissolution of polymer tablets. In vivo the tablets will encounter a variety of different environments with different ionic strengths and amphiphilic content [6, 18]. Moreover, the composition of the environment varies over time depending on food intake, so-called fasted or fed states, and also between different individuals. Today there are few methods available to address these problematic effects. Attempts to address the issues include studies of various lipid-based formulations [19]. For instance, Woo et al [20] showed that effects of food intake could be decreased via a self-emulsifying formulation. Nevertheless, there is still a lack of formulation approaches for conventional eroding tablets that aim to minimize food effects on the formulation. There are additional problems linked to fasted fed state that are cannot be addressed by formulation strategies for eroding tablets such as the variation of emptying rate of the tablets from the stomach. This might affect drugs that have different uptake in different parts of the GI-tract. However, lipophilic substances that do not change charge during passage in the GI tract are less susceptible to empting rate.

During our previous investigations of Pemulen TR2 tablets we have found that the release of hydrophobic substances is affected by the presence of surfactants and/or the charge of the polymer backbone, i.e. pH or ionic strength [7, 8]. These factors are directly relevant for the different conditions encountered in the gastrointestinal tract. It was also established that the effects on the release of the drug were correlated to the behaviour of the polymer, and specifically the gel layer. In pure water, the polymer showed only minor swelling and applying shear to the tablets lead to comparatively rapid disintegration and release of non-soluble tablet/polymer particles, which increased the release rate [7]. Addition of buffer to the medium and/or surfactant at concentrations above the CMC increased the swelling. With surfactant the polymer became fully soluble and could swell indefinitely. Consequently, a thicker gel layer was formed during dissolution, and a slower release was observed. The swelling was further evaluated via NMR Chemical Shift Imaging where clear differences were seen in the concentration profiles of the polymer during dissolution correlated to the polymer solubility and phase behaviour [8]. The results indicate that the release of hydrophobic substances from Pemulen TR2 could potentially be affected by differences in the intestinal fluid between fasted or fed states. However, we also found that by adding sufficient amounts of surfactant to the tablet formulation the effects from differences in the release medium could be circumvented [7]. Consequently, addition of surfactant could be used as a possible approach to avoid problematic food effects.

This paper further investigates the potential of Pemulen TR2 tablet formulations with hydrophobic substances. Griseofulvin is used as model substance and the release is studied in simulated intestinal fluid (SIF) in accordance to references [6, 21, 22]. The focus is on the effects of the differences in amphiphilic content in the media. In addition, the study investigates whether it is possible to circumvent the effects of amphiphilic substances in the external medium via addition of an amphiphilic substance to the formulation. Here sodium docecyl sulfate (SDS) is used as a model amphiphilic substance. SDS is a well-known and studied anionic surfactant, which is also approved for oral formulations. SDS was also chosen, as it is substantially different from the surfactants in bile and thus would give an indication if formation of mixed miscells would affect the release profile. As in our previous studies, the tablets in this study contain a high amount of polymer to avoid possible experimental variations due to tablet inhomogeneities. This leads to total dissolution times that are too extended to be relevant for commercial tablets. However, the aim here is to evaluate the presence or absence of a susceptibility to food effects, not to optimise the release time. To further investigate effects from the content in the intestinal fluid and in order to gain additional molecular insight into such effects, rheological measurements are done on model polymer solutions. Dissolution experiments are done both with biorelevant media and with SDS solution. The later is done to see if there is a difference in dissolution when the media contains the same amphiphilic substance as in the tablets compared to using more biorelevant surfactants.

## Materials and Methods

### Chemicals

#### Dissolution experiments

The following chemicals were used for the tablet dissolution experiments. Pemulen TR2 NF was kindly provided by Lubrizol Advanced Materials (Brussels, Belgium). According to the supplier, the polymer consists of poly (acrylic acid) cross-linked with allylpentaerytritol and contains 52–62 wt% of carboxylic acid groups. Pemulen is hydrophobically modified with grafted C10-C30 alkyl-chains. Griseofulvin was purchased from Sigma (Steinheim, Germany) and used as provided.

Sodium dodecyl sulphate (SDS) was purchased from VWR International (Poole, England). The CMC for SDS is 8.3 mM in pure water [23] and was determined, with a tensiometer and a de Noüy ring, to 1.8 mM at 37°C in 0.1 M phosphate buffered solution, pH 7.2. For tableting, lactose monohydrate from Merck (Darmstadt, Germany) was used as supplied and talc and magnesium stearate were of analytical grade.

The following chemicals were used to prepare the simulated intestinal fluid. Sodium taurocholate (NaTC) was purchased from TCI (Zwijndrecht, Belgium). Lecithin from soybeans and sodium oleate was acquired from VWR International (Leuven, Belgium and Fonteray sous Bois, France). Glycerol monooleate was purchased from Grindsted Products (Copenhagen, Denmark) and maleic acid was acquired from Sigma (Steinheim, Germany). NaOH and NaCl were of analytical grade. All chemicals were used as received.

#### Model gel systems

Pemulen TR1 and Carbopol 981 were supplied from Noveon Inc (Cleveland, Ohio US) and Pemulen TR2 from BFGoodrich Co (Cleveland, Ohio US). Pemulen TR1 and Pemulen TR2 contain both 52–62 wt% COOH groups, whereas Carbopol 981 contains 56–68 wt%. Pemulen TR2 contains a larger amount of hydrophobic groups than Pemulen TR1, as confirmed by 1H-NMR measurements of the polymers dissolved in deuterated DMSO.

For the model gel systems with water added amphiphiles the following chemicals were used: Sodium taurocholate (NaTC) purchased from Prodotti Chimici e Alimentari S.p.A (Basaluzzo, Italy) and SDS purchased from Merck (Darmstadt, Germany). The lecithin used was Lipoid E PC, phosphatidyl choline from egg lecithin, purchased from Lipoid GmBH (Cologne, Germany). Preparation of SIF media for rheology experiments were made with the same chemical as for the dissolution experiments.

### Dissolution experiments

#### Tablet preparation

Tablets were prepared with compositions as specified in Table 1. The manufacturing procedure for all tablet compositions was the same as in previous studies [7]. The amount of griseofulvin was chosen in order to maintain sink conditions during dissolution. First lactose, griseofulvin, polymer and SDS (if applicable) were mixed in an intensive mixer (Kitchen Aid) for 5 min. A (70:30) mixture of ethanol (99.5%) and 0.001 M aqueous HCl was used as granulation fluid. The granulation process started with gently spraying granulation fluid over the powder, with interruptions to allow the fluid to absorb. The procedure was continued until the desired properties of the granulation were achieved. The granules were then dried at 50°C during 2 h and subsequently sieved through a 2 mm sieve. In cases where the granules were too large, a food processer (Philips Cucina) was used to further reduce particle size. The granulation was then lubricated by mixing with talc and magnesium stearate using a cone-blender for 2 min after addition of each component.

**Table 1:** Composition of tablets used in this study.

	Formulation 1	Formulation 2
Griseofulvin (wt%)	1	1
Pemulen TR 2 (wt%)	30	30
Lactose (wt%)	67	59.5
Mg-stearate (wt%)	1	1
Talc (wt%)	1	1
SDS (wt%)	0	7.5

Tablets were prepared with a single-punch tableting machine (Diaf type TM20, Denmark). Friability and hardness test, in accordance with US pharmacopeia, were performed afterwards to confirm good physical properties of the produced tablets. All tablet used achieved a friability less then 2% and a hardness above 8 kp (above 5 kp no effects of hardness on the dissolution have been seen).

#### Preparation of bio-relevant media

The compositions of the simulated intestinal fluids (SIF) are summarized in Table 2. A phosphate buffer solution was used to study the behavior of the tablets without any amphiphilic compound added to the dissolution medium.

**Table 2:** Compositions of dissolution medium.

	FaSSIF	FeSSIF
Sodium taurocholate (mM)	3	10
Lecithin (mM)	0.2	2
Glycerol monooleate (mM) (GMO)	-	5
Sodium oleate (mM) (SO)	-	0.8
Maleic acid (mM)	19.12	55.02
NaOH (mM)	34.8	81.65
NaCl (mM)	68.62	125.5
pH (qs.)	6.5	5.8

The solution pH was then adjusted to 6.5 (FaSSIF) or 5.8 (FeSSIF). After the last pH adjustment the volume was adjusted by adding deionized water to 1 l.

#### Dissolution test

Dissolution experiments were carried out at 37°C in a USP dissolution apparatus (Prolabo Intelligent dissolution tester Novakemilab, Sweden) with rotating discs at a paddle speed of 100 rpm. To the USP vessels 1 l of dissolution medium was added. The discs were made of stainless steel and had a diameter of 50 mm and thickness of 3 mm. The discs had indentations, 1 mm deep and 12 mm in diameter, at the centre to simplify mounting the tablets. Tablets were weighed prior to experiment and glued to the discs with Scotch^®^ contact adhesive (3M, Glostrup, Denmark).

Aliquots (1 ml) were manually withdrawn at specified time intervals in order to analyse the release of griseofulvin. The samples were analysed by HPLC (Hewlett-Packard, HP Series 1050)with a uv-vis decetctor (290 nm) using a flow of 0.4 mL/min on a reversed phase Acclaim RLSC C18 2.2 μm, 120 Å, 2.1 x 50 mm column. A gradient was used with acetonitrile (AcN) and 0.1% acetic acid (Table 3). Samples were mixed with AcN prior to analysis, for samples containing a low amount of drug a 25/75 mixture was made and for higher concentrations (at later times) a 50/50 mixture. The released amount (%released) was calculated according to Eq 1.

$$\%released=\frac{\left(c_{g}\times \left(V_{0}-V_{a}\times \left(n_{a}-1\right)\right)\right)+\Sigma_{n=0}^{n_{a}-1}\left(V_{a}\times c_{g,n}\right)}{m_{g}}$$ (1)

**Table 3:** Gradient used during HPLC analysis.

time / min	AcN
0–1	30%
1–5	30 to 50%
5–6	50%
6–7	50 to 30%
7–12	30%

Here, the concentration of griseofulvin according to HPLC is given by c_g_ and the initial volume of the vessel is V_0_ (equal to 1 L). V_a_ and c_g,n_ denote the volume and the concentration in the samples, respectively, and the number of samples is denoted n_a_. The amount of griseofulvin in a tablet is given by mg.

### Rheology measurement of model gels

#### Sample preparation

Simplified polymer mixtures in water with addition of amphiphiles were prepared by dry mixing of appropriate amounts of polymer and surfactant in sample tubes, followed by addition of solvent to yield a concentration of 1 wt% of polymer. The sample tubes were sealed with a screw cap and put on tilt table for a couple of days. Mixing of the samples was accomplished by centrifugation several times and with agitation in between. The pH in the homogenous samples was measured with a pH electrode and was adjusted to 5.8–6.0 by means of drop-wise addition of 1 M or 2 M NaOH. Care was taken to minimize the volume of added NaOH and the sample concentrations were recalculated to account for the volume change. Samples in SIF media with and without SDS were prepared by first weighing the polymer and (when applicable) SDS, and then adding the appropriate medium. SDS was added at the same surfactant:polymer weight ratio (7.5:30) as in the tablets. This resulted in a concentration of ca. 8 mM SDS in the solution, which is similar to cmc in water and well above cmc in a complex buffer as the one used here. The samples were put on tilt table and were centrifuged and agitated until homogeneous as determined by visual inspection. The pH of the homogeneous gels was measured and set to 7.2 (buffer), 6.5 (FaSSIF) or 5.8 (FeSSIF) with addition of 2 M NaOH. The concentrations in the samples were recalculated to account for the volume change. After the appropriate pH was achieved, all samples were kept in a refrigerator to avoid microbial growth. All samples were taken out and kept at room temperature one day before analyses and measurements.

#### Oscillation rheology measurements

The rheology measurements in surfactant water solutions were performed with a controlled stress rheometer (Reologica StressTech). A plate-plate symmetry with a radius of 1.5 cm was used with a gap of 1 mm. The temperature was 20°C. The elastic (*G*’) and viscous (*G*”) moduli were measured in a frequency sweep between 0.01 and 10 Hz at a constant stress of 1 Pa for stiff gels and 0.2 Pa for viscous liquids. All measurements were performed in the linear viscoelastic region, as confirmed by performing a subsequent stress sweep between 0.07 and 100 Pa at 0.2 Hz.

Measurements on gels in SIF and in buffered solutions were carried out in a controlled stress rheometer (Malvern Kinexus), with serrated plate-plate geometry (radius of 4 cm) and a gap of 1 mm. The temperature was 20°C. The elastic (*G*’) and viscous (*G*”) moduli were measured in a frequency sweep between 0.01 and 10 Hz. The constant stress was evaluated by the rheometer via performing an initial stress sweep between 0.07 and 100 Pa at 0.2 Hz to locate the linear viscoelastic range.

## Results and Discussion

### Rheology of model polymer and polymer/surfactant samples

#### Characterisation of polymer dispersions and solutions

The rheological measurements were performed on two hydrophobically modified polymers, Pemulen TR1 and TR2, with different degrees of hydrophobic modification. The effects of hydrophobic modification were also evaluated via measuring the rheology of non-modified poly (acrylic acid), i.e. Carbopol 981.

All polymer samples formed acidic dispersions in pure water. At concentrations below 0.05 wt%, the dispersions were transparent and non-viscous and at higher concentrations they became notably viscous. At around 1 wt% the mixtures were opalescent and thick, and at concentrations above ca. 5 wt%, stiff white gels were formed. If the pH of the systems was adjusted so that alkaline conditions were obtained, the thickening effect of the polymer appeared at much lower concentrations and the mixtures were transparent, stiff and did not flow when tilted. The effects of pH and concentration are expected. By titrating the polymer carboxylic acid groups (i.e. adding charge) the solubility is increased and the polymer becomes more extended and swells, hence building viscosity [24, 25]. However, the presence of hydrophobes in Pemulen adds another layer of complexity. In water the hydrophobes of hydrophobically modified polymers typically aggregate, resulting in an increased viscosity and, also, a decreased water solubility [26]. In the present study the focus was on comparing different aqueous polymer dispersions and solutions. The concentration was therefore kept at 1 wt% throughout and the pH was set to 6.

**Fig 1:**
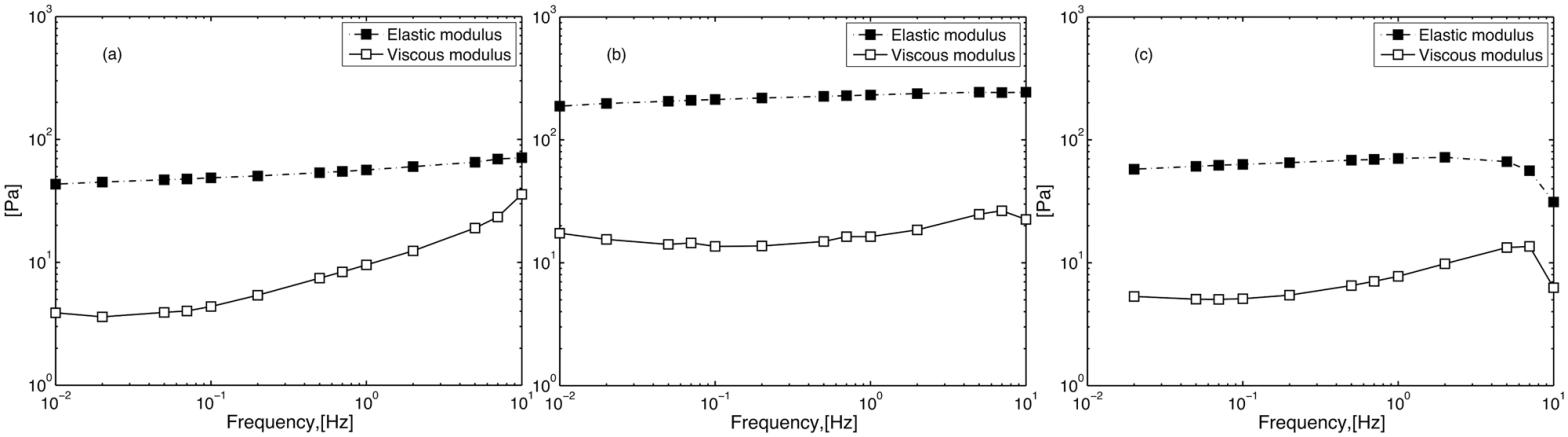
Oscillation measurements at constant stress (1 Pa) of 1 wt% polymer gels in water at pH 6. From left to right Carbopol 981 (a), Pemulen TR 1 (b) and Pemulen TR 2 (c). The elastic (G’, filled squares) and viscous (G”, open squares) moduli are shown as functions of frequency.

Fig 1 show typical frequency sweeps of 1 wt% Pemulen TR1, Pemulen TR2 and Carbopol 981 in pure water at pH 6. In all cases, G’ is much higher than G” and shows no, or only minor, frequency dependence. This rheological fingerprint is typical for a gel. Pemulen TR1 has the highest G’ and G” values, whereas Pemulen TR 2, with its higher hydrophobicity, has lower G’ and G” values. The rheology of Carbopol 981 was similar to that of Pemulen TR2, while Pemulen TR1 gave a much stiffer gel.

#### Simple mixtures with surfactant

**Fig 2:**
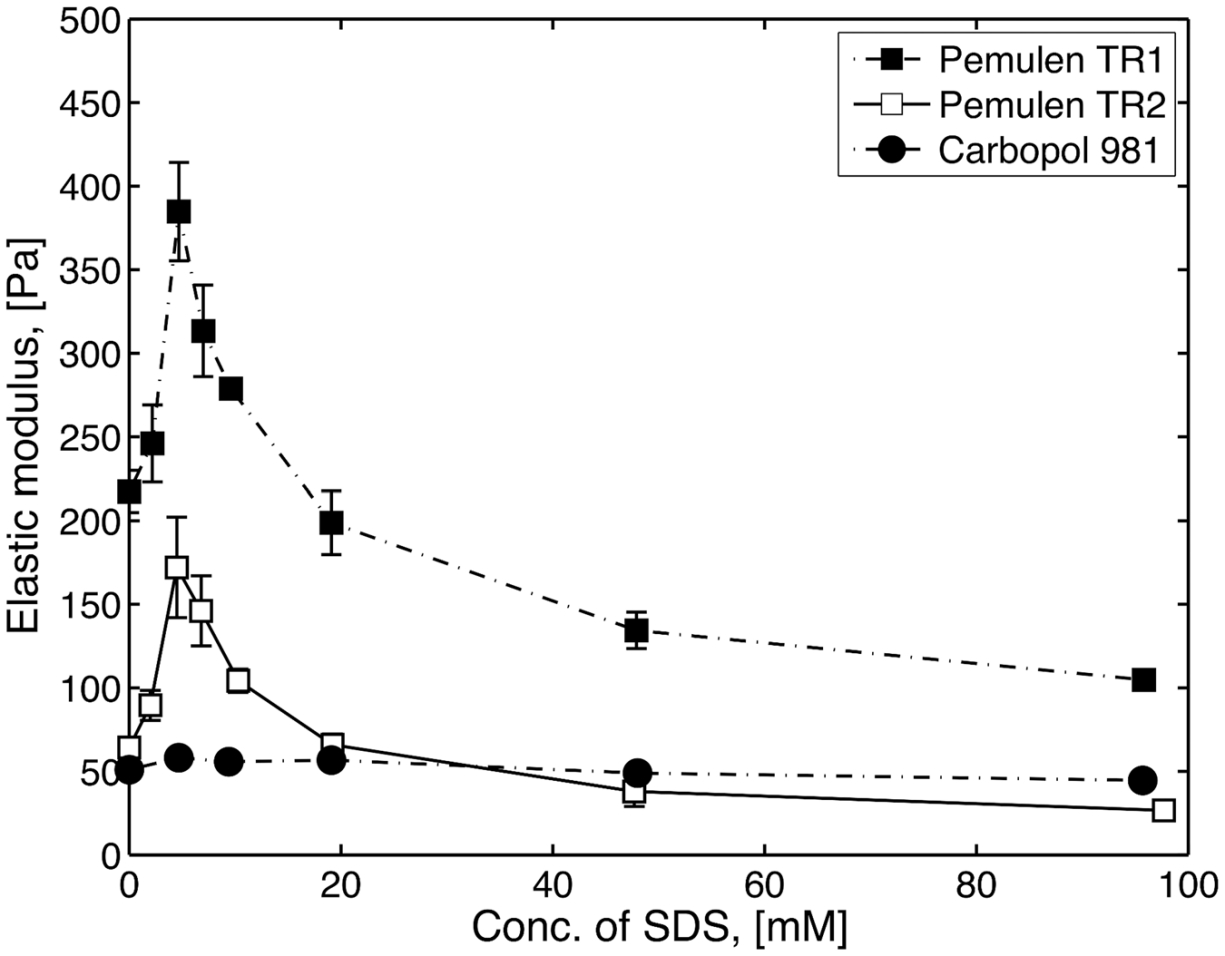
The elastic modulus (G’) at 0.2 Hz as a function of the SDS concentration for 1 wt% Pemulen TR1 (filled square), TR2 (open squares) and Carbopol 981 (filled circles). The data points represent the mean values of three samples and error bars show the standard deviation (n = 3).

**Fig 3:**
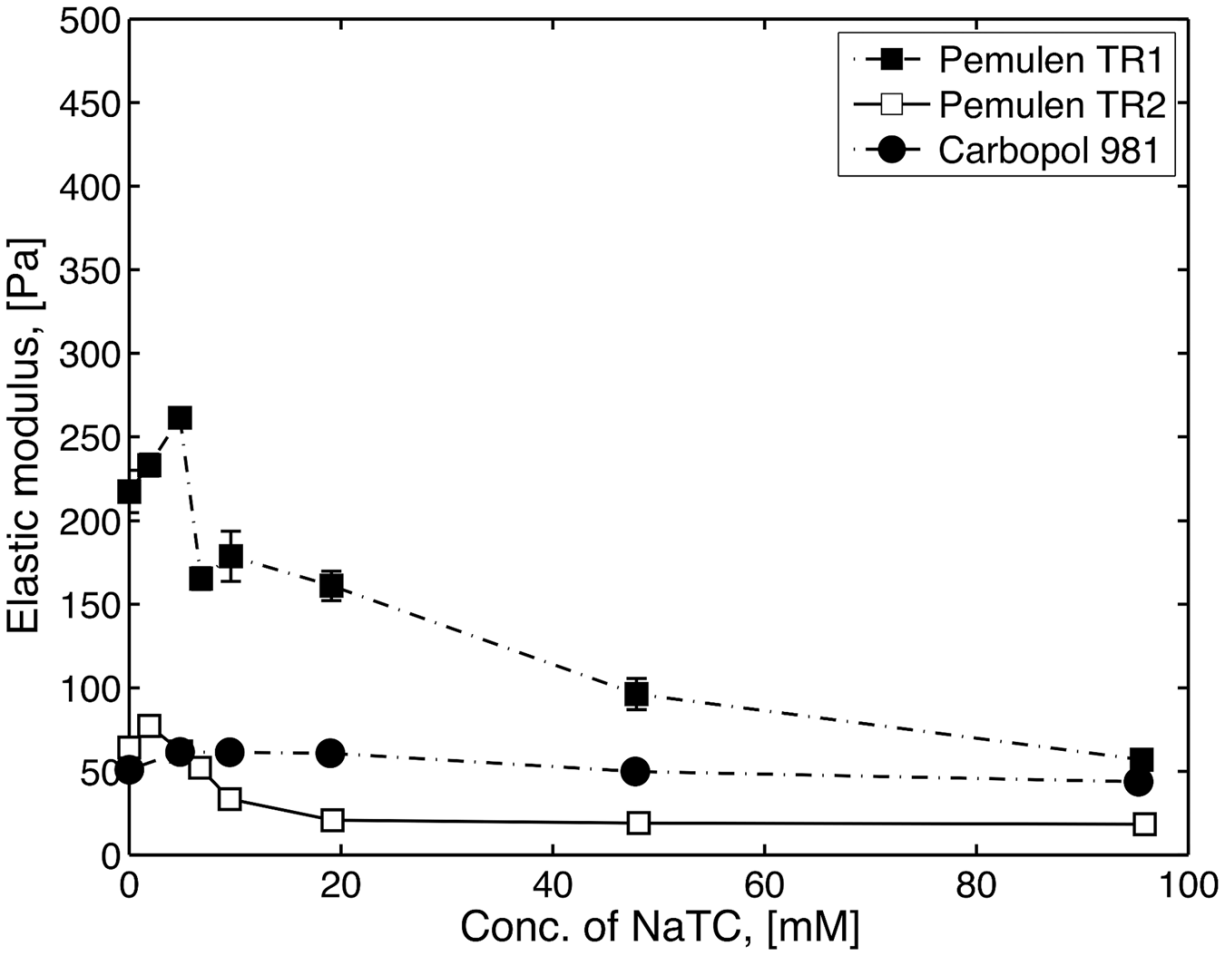
The elastic modulus (G’) at 0.2 Hz as a function of the NaTC concentration of for 1 wt% Pemulen TR1 (filled square), TR2 (open squares) and Carbopol 981 (filled circles). The data points represent the mean values of three samples and error bars show the standard deviation (n = 3).

In order to investigate possible effects of surfactants on the rheology of the polymer in the intestinal fluid, simplified media containing NaTC and lecithin were investigated. Furthermore, SDS was used as reference surfactant, also allowing comparisons with previous results [7, 27]. Figs 2 and 3 show the elastic modulus, G’ (at 0.2 Hz), as a function of the concentration of SDS or NaTC for Pemulen TR1, Pemulen TR2 and Carbopol 981. The G’ value without additives (i.e. at a surfactant concentration cs = 0), will henceforth be referred to as G’0. The behaviour on increasing surfactant concentration is similar to that already seen for similar materials (Pemulen TR2 and Carbopol 934) with addition of non-ionic surfactants [28]. For HMPAA (Pemulen TR1 and TR2) solutions, both NaTC and SDS gave rise to a maximum in G’, albeit at slightly different concentrations for the two polymers, due to their different degrees of hydrophobic modification. Carbopol, on the other hand, was nearly unaffected by the addition of the surfactants. For SDS, the G’ maximum appeared at concentrations close to the CMC. It is well known that upon addition of surfactant to a HM-polymer solution, the surfactant associates with the hydrophobes of the polymer and forms mixed aggregates [27, 29]. At low concentrations, the interactions between surfactant molecules and hydrophobes from different strands strengthen the gel network and the viscosity increases. However, further increase of the concentration of surfactant eventually leads to solubilisation of the hydrophobes in surfactant micelles, so that the hydrophobic crosslinking of polymer chains is lost. In this regime, G’ decreases to a value below G’0. No G’ maximum was observed for Carbopol 981 on addition of surfactant, consistent with the lack of hydrophobes in this polymer.

Compared to SDS, NaTC gave a smaller increase of G’ upon increasing concentration. Furthermore, the post-maximum decrease of G’ was observed to commence at a slightly lower amphiphile concentration. Unlike SDS, NaTC does not form spherical micelles but forms poorly defined, polydisperse aggregates with only a few molecules in each assembly [30, 31]. Presumably, added NaTC only marginally strengthened the hydrophobic domains in the HM-polymer, instead they soon became effectively solubilized by the small aggregates.

**Fig 4:**
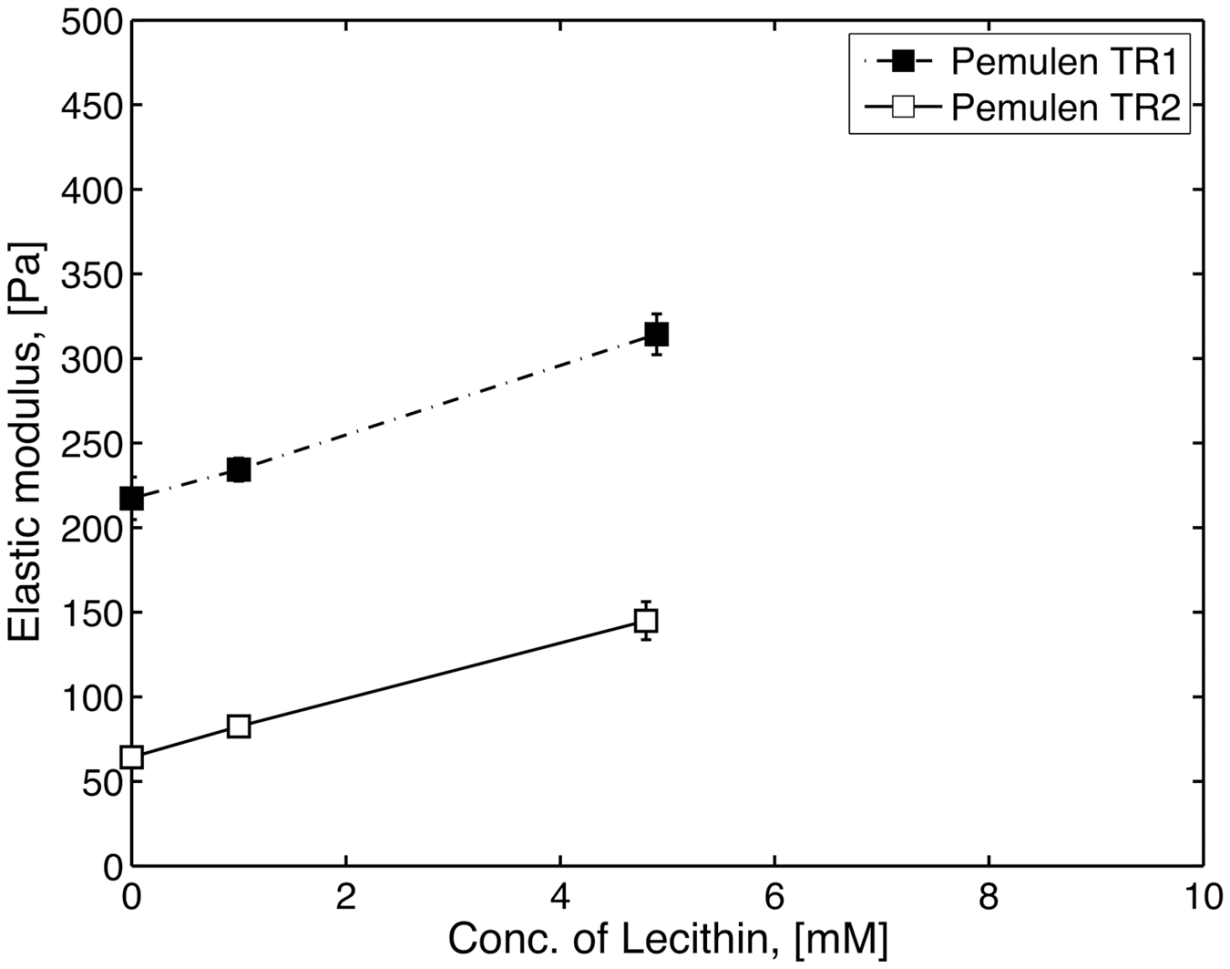
The measure elastic modulus (G’) at 0.2 Hz for homogeneous samples of polymer and lecithin, for 1 wt% Pemulen TR1 (filled square), TR2 (open squares). At lecithin concentrations above 5 mM the systems were not homogenous and were thus not subjected to further characterization. Carbopol, which lacks hydrophobes, formed heterogeneous samples at all concentrations with lecithin. The data points represent the mean values of three samples and error bars show the standard deviation (n = 3).

Mixtures with lecithin and Pemulen TR1 formed turbid, but macroscopically homogenous gels up to lecithin concentrations of ca. 5 mM. At higher concentrations, large, white lumps of lecithin were formed in the gel. The more hydrophobic material Pemulen TR2 formed a weakly turbid gel up to 5 mM lecithin. At higher concentrations, small white lumps could be observed in the gelatinous solution. The differences in turbidity may be correlated to a higher solubilising capacity of Pemulen TR2 towards lecithin where lecithin presumably was solubilised in the hydrophobic regions formed by association of the hydrophobes. In agreement with this idea, Carbopol 981 did not dissolve any lecithin and white lumps of lecithin were formed in the transparent gels already at low concentrations (1 mM). Rheological measurements were made on the homogenous samples containing lecithin and small amounts of lecithin improved the elastic properties of the HMPAA solutions in a similar manner as SDS and NaTC (Fig 4).

These initial rheological studies indicated that at no added surfactants and at high amount of added surfactants Pemulen TR-2 had a rheological behaviour similar to that of Carbopol while Pemulen TR-1 showed stiffer gels. Thus Pemulen TR2 was chosen for the consecutive studies.

#### Pemulen TR2 in biorelevant dissolution media

In order to be able to compare the release behaviour in the dissolution experiments with the rheological behaviour of the polymer, the rheology was also measured for model gels in complex media. This was done only for the more hydrophobic Pemulen TR2, as this was the polymer used for production of tablets, both in the present study and in the previous ones [7, 9].

**Fig 5:**
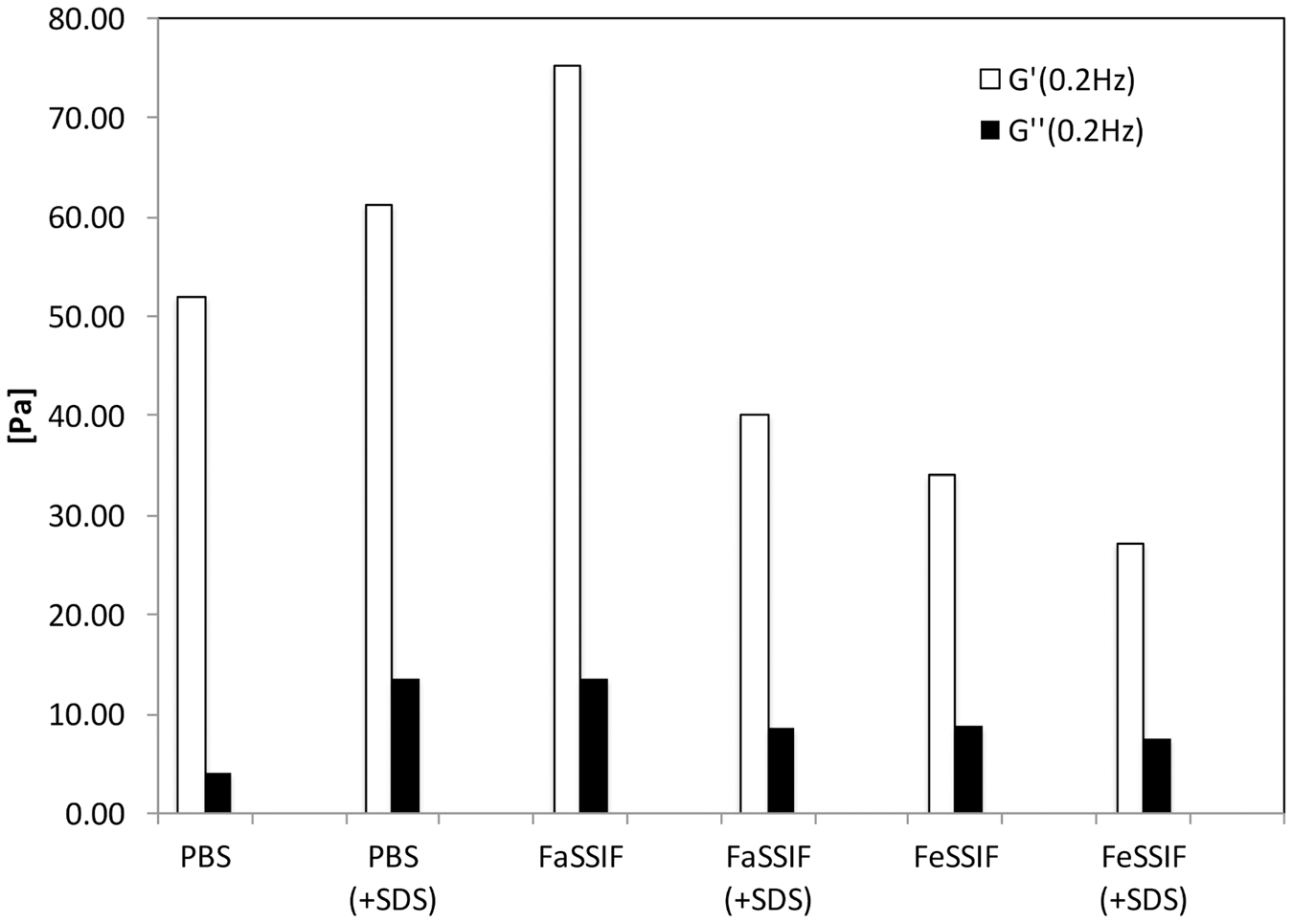
The elastic (G’, black bars) and viscous (G”, hatched bars) moduli at 0.2 Hz shown for 1 wt% model gels in dissolution media with (+SDS) and without SDS in the gels. The SDS concentration was ca. 8 mM. The data points represent the mean values of two measurements and error bars show the standard deviation (n = 2).

All gels formed in the dissolution media were highly viscous and did not flow visibly when tilted. Clear gels were formed in buffered solution and FaSSIF, whereas gels in FeSSIF were slightly turbid. This agrees with the results and discussion above and it can be hypothesized that NaTC and lecithin formed large mixed aggregates in the gel, which scatter light. Interestingly, if SDS was added to the gels (in a similar ratio as in the tablets, see below) the turbidity of the FeSSIF gels decreased, probably due to solubilisation of lecithin. As can be seen the rheology of FaSSIF and FeSSIF gels differed significantly but when SDS is added this variation is considerably decreased, Fig 5.

As would be expected from their visual appearances, all gels displayed higher G’ than G” in all media. The highest values were seen for pure gels in FaSSIF and gels with SDS in buffered solution, whereas the lowest values were seen in FeSSIF with SDS. This can be explained from the behaviour of the simple mixtures and the association of surfactant and hydrophobes. In FaSSIF and in PBS (+SDS) the concentration of amphiphiles was low (3.2 and 8 mM respectively) and close to the surfactant concentration at maximum, c_s,max_, of the simple mixtures. The surfactants stabilized the hydrophobically associated cross-links and the viscosity increased. The surfactant concentration in the FeSSIF solution (12 mM) was much higher than the c_s,max_ in the simple mixtures. Therefore, the amount of surfactant was large enough to completely dissolve the cross-links in the dynamic polymer network. This also true for FaSSIF (+SDS) where the addition of SDS gave a concentration of amphiphiles equivalent to the FeSSIF mixtures (ca. 11 mM).

### Release studies

**Fig 6:**
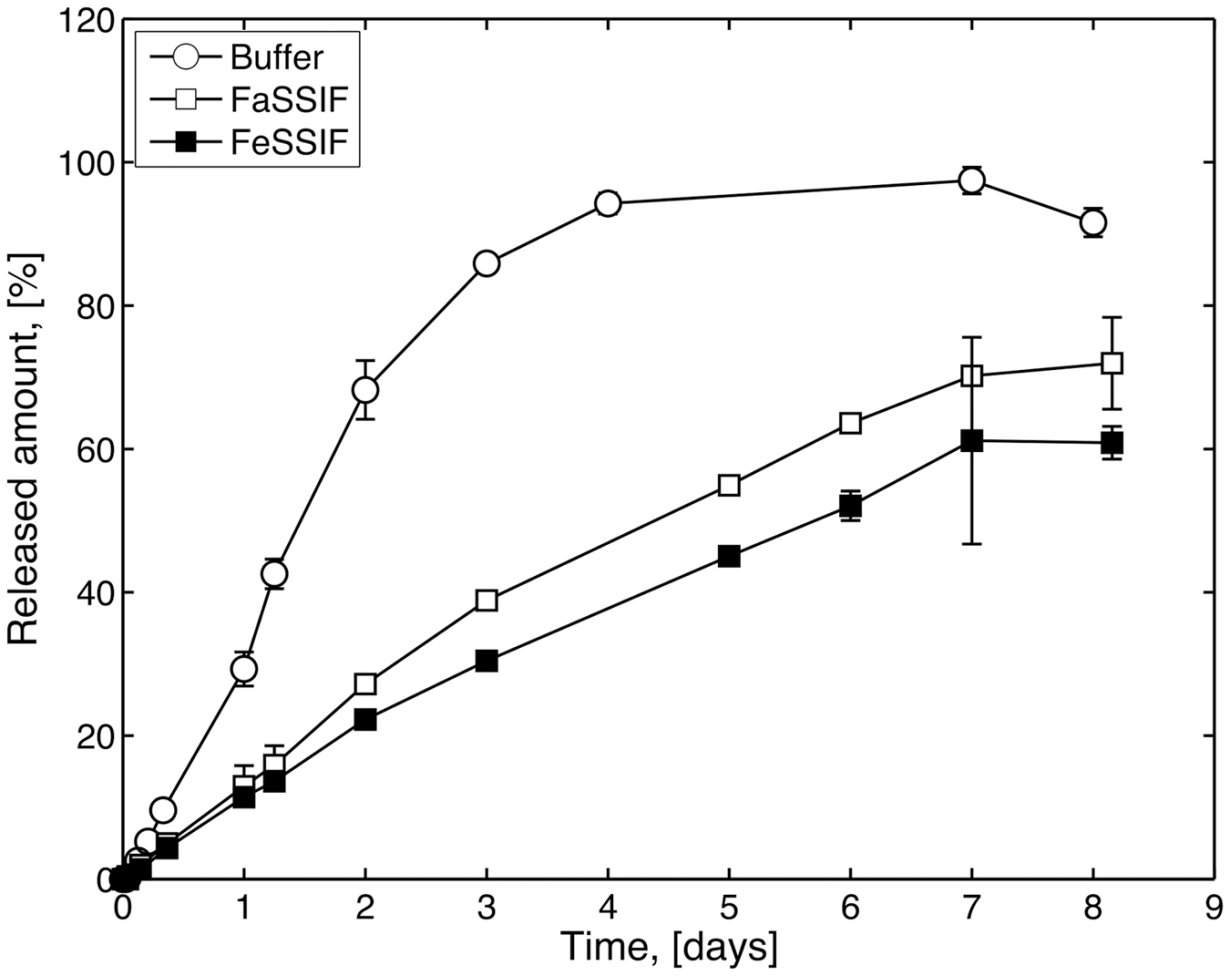
Release of griseofulvin from tablet without SDS (Formulation 1) in buffer (open circles), FaSSIF (open square) and FeSSIF (filled square). Error bars represent standard deviation (n = 2).

The release of griseofulvin from Pemulen TR2 tablets without added surfactant (Formulation 1) is shown in Fig 6 for tablets dissolving in buffer and SIF solutions. The tablets generally showed a slow and linear release rate, which is retained throughout the experimental period. None of the tablets were dissolved after eight days. Tablets dissolving in SIF media showed an even slower release than in buffer and clear differences can be established between the tablets in the various media. The slowest release was in FeSSIF solution. The tablets in SIF were not fully dissolved during the experimental period. However, after four days the SIF solutions became more and more turbid, and at after even longer dissolution the solutions turned milky. Hence, the solutions were not considered stable throughout the extended experimental time and the experiments were ended before complete dissolution.

The release of griseofulvin from the tablets (Formulation 1) was similar to the release previously established for ibuprofen in a similar formulation [7]. Ibuprofen is a hydrophobic drug, but it was not considered poorly soluble during the previous studies. Both ibuprofen and griseofulvin was studied at concentrations below sink conditions. Thus, technically below the limit where the tablets as such are considered to poorly soluble. However, the concentration of griseofulvin in the tablet was above the solubility limit for the majority of the investigation (giving a constant concentration of dissolved griseofulvin in the tablet during the experiment) while the concentration of ibuprofen could not be guarantied to be this especially as solubility of ibuprofen will be affected by the local pH in the tablet. Both ibuprofen and griseofulvin can be assumed to interact with the polymer and the added surfactants via hydrophobic interactions. During our previous studies we could see that the release of ibuprofen was correlated to the erosion and the behaviour of the polymer. The similar release of griseofulvin indicates an equivalent behaviour. Furthermore, preliminary results with ibuprofen in SIF media show similar release characteristics as for griseofulvin.

There were large differences in the behaviour between the tablets in the various media, as established visually by examining the tablets during dissolution in the bath. Tablets in pure buffer formed thin but clear gel layers during dilution, whereas tablets in SIF formed thicker, non-transparent gel layers. In addition, eroded tablet particles could be seen in the buffered solution. This behaviour is in accordance with the properties of the model gels in SIF solutions, which indicated that large aggregates of NaTC and lecithin were formed in the turbid gel layers of the dissolving tablets.

**Fig 7:**
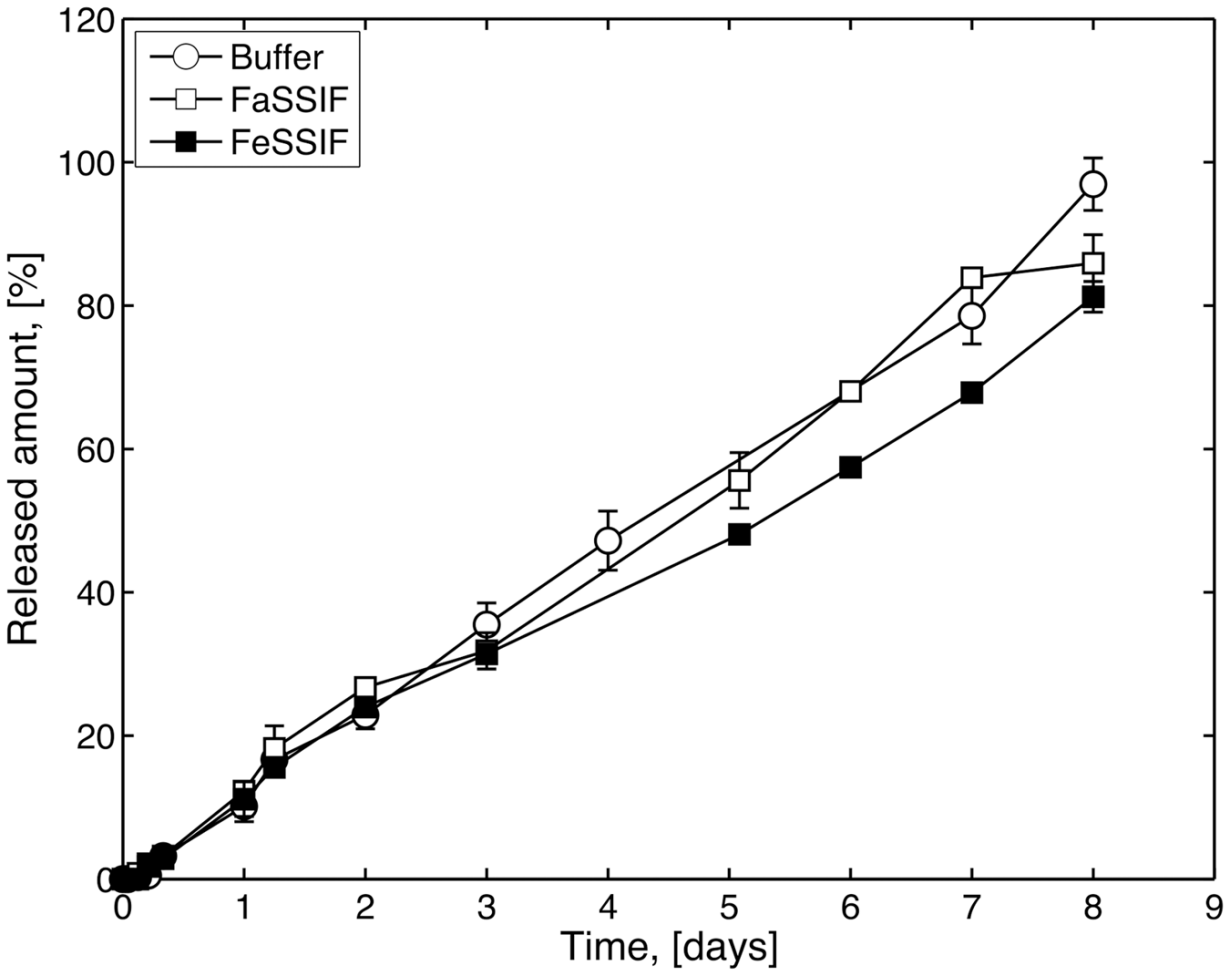
Release of griseofulvin from tablets containing SDS (Formulation 2) in buffer buffer (open circles), FaSSIF (open square) and FeSSIF (filled square). Error bars represent standard deviation (n = 2).

Tablets containing SDS (Formulation 2) behaved differently in many key aspects, see Fig 7. Most importantly, the differences in release profile between the different media were lost and the tablets of Formulation 2 showed similar release rates and profiles in all media. Examining the tablets visually in the bath during dissolution showed that all tablets formed thick gel layers and only small amounts of eroded tablet particles could be seen in the baths. None of the tablets were dissolved completely during the experimental time. However, due to the physical instability of the SIF solutions (see above) the experiments were terminated after eight days. Due to there composition SIF and FASSIF is not fully stable for eight days but the experiments were followed as long as possible to see if any changes from the zero order profile could be observed.

The release rate from the various tablets in the different media were quantified as the slope of the release profiles between 5–80% released amount, and the results are shown in Table 4. The calculated release rates further confirm the behaviour of the tablet and only minor differences can be established for tablets with SDS dissolving in buffer and SIF solutions. Tablets without SDS (Formulation 1) in SIF also showed a similar release as tablets with SDS (Formulation 2). The most rapid release rate was seen for Formulation 1 in buffer which was almost three times faster than the remaining releases.

**Table 4:** Release of griseofulvin from HMPAA tablets with and without SDS in buffer, FaSSIF and FeSSIF. Release rate is calculated as the slope of the release profile (Figs 5 and 6).

Media	Formulation 1 [% /day]	Formulation 2 [% /day
Buffer	30.39±0.79 (*n* = 3)	13.39±0.65 (*n* = 3)
FaSSIF	10.71±0.20 (*n* = 2)	12.89±0.96 (*n* = 2)
FeSSIF	8.72±0.32 (*n* = 2)	10.43±0.28 (*n* = 2)

Clearly the release from HMPAA tablets is affected by the presence of amphiphilic compounds and the amphiphilic content in SIF affected the release, which is slowed down. From the studies of model gel systems it is also clear that the behaviour of Pemulen TR2 is greatly affected by pH and amphiphilic content. The measured rheology and the visual appearance of the samples showed that the properties of the polymer solutions varied greatly with changes of pH and/or addition of surfactant. Nevertheless, the mixtures investigated by rheology are not directly comparable to the dissolving tablets. In fact, we have seen in previous unpublished studies that the release in a non-surfactant system from TR1 (high viscosity) is faster than from TR2. Moreover, studies that used NMR Chemical Shift Imaging to obtain concentration profiles in the swollen tablets in surfactant solutions has showed a rapid decrease of the surfactant concentration in the gel layer. Hence, the concentration in the gel layer is most likely different from the concentration in the surrounding medium and the investigated rheology samples. The same studies also showed that a majority of the surfactant molecules would reside in mixed aggregates with the hydrophobes. Nevertheless, the rheology results are useful since they shed light on the interactions between surfactants and polymer in the systems.

The amphiphilic compounds in the SIF media aggregated on the hydrophobes and solubilize the polymer, in a similar manner as reported previously [7, 8]. The aggregation was confirmed in the rheology measurements, where a clear increase in viscosity is seen. As a result of the interaction the soluble polymer formed a thicker gel layer and the erosion of the tablets is slowed down. Poorly soluble drugs are typically released via erosion of the tablet, and the drug release is thus directly determined by the behaviour of the polymer [32, 33]. When the erosion is slowed down the release of griseofulvin is also decreased [7]. This could either be due to the slower erosion itself, or there could be a shift in release mechanism so that the release is gradually more determined by the interaction of griseofulvin with the polymer/surfactant aggregates. The effects on the swelling of the tablets are closely related to those seen in previous experiments with the same system, where we have been able to visualize the solubilization effect using NMR [8]. In some cases non-transparent gel layers were formed, probably due to formation of large aggregates of mixed micelles of NaTC and lecithin. Through incorporation of a sufficient amount of a surfactant (SDS) in the tablets the effects could be circumvented and tablets that were insensitive to the amphiphilic content in the external media were obtained. The surfactant in the tablets aggregated on the hydrophobes and solubilized the hydrophobes eliminating the solubilisation effect from amphiphiles in the surrounding medium and the already slow release was maintained.

When the erosion is slowed down the dominant release mechanism becomes slower and a slower release is seen. Furthermore, NMR studies shows that the transport of the drug is very slow in the gel layer and that a majority of the drug molecules will be solubilized in the hydrophobic domains formed by the hydrophobes and/or the amphiphiles. These also showed that the micelles will predominately be associated with the hydrophobes already at low polymer concentrations, thus a majority of the drug molecules will be transported with the polymer as this expands, similar to the process of translocation [34–36]. To some extent this also explains why we see an effect from addition of surfactant to the tablets and why the surfactant was not quickly transported out. The transport of surfactant out from the tablet becomes very slow owing to the fact that a majority of the surfactant molecules are associated with the hydrophobes.

The release shown in the present work is very slow and obviously needs to be more accelerated if the systems are to be progressed as candidates for pharmaceutical development. The tablets were intentionally prepared with a high polymer content, in order to prevent effects of inhomogeneity, facilitate building of fundamental understanding, and to manifest discrepancies. Preliminary experiments show that decreasing the amount of polymer decreases the dissolution time while maintaining the linear profiles; this is of course something that needs to be further scrutinized and exploited. It is also possible to decrease the dissolution time by decreasing the amount of hydrophobes on the polymer [7]. The benefit of such a formulation is that it could combine controlled release with a formulation that affects the apparent solubility of the low soluble substance by addition of surfactants. As seen by the work of Tabandeh and Mortazavi [10] mixing Carbopol and Pemulen polymers could be an additional way to tune the release profile.

## Conclusions

We have previously investigated the potential of HMPAA to be used in oral controlled release formulations. The polymer has shown promising results with hydrophobic substances, an increasing issue in pharmaceutical development. During the investigations it was found that HMPAA tablets were sensitive to amphiphilic compounds in the surrounding medium, which could potentially aggravate problems in vivo due to the differences in amphiphilic content between fasted or fed states. In this paper we further validated the potential of the polymer and the possibility that via incorporating a sufficient amount of surfactant in the tablets food effects, e.g. effects of amphiphilic compounds, could be circumvented. When adding surfactant to the tablets, no difference in the release of a model hydrophobic substance was seen for three different in vivo related media. It was also shown, by investigations of the rheological behaviour of model gels, that the differences and the release behaviour could be correlated to the interactions between the hydrophobes on the HM-polymer and the added amphiphilic compounds. Compared to the release in the absence of amphiphiles the release was slower and the tablets formed larger gel layers. In order for HMPAA to be a realistic candidate for tablet formulations the release times need to be decreased and preliminary experiments have shown that a decreased dissolution time can indeed be achieved via decreasing the polymer content. To conclude, hydrophobically modified cross-linked poly (acrylic acid) polymers have a potential to be used in oral controlled release formulations and may be used as the basis for formulations that are less sensitive to food effects than current technology. However, in order to obtain a product with a clinical relevant release profile the tablets and possibly the polymer has to be optimised to obtain a faster release profile. There will also have to be further studies on how the tablets behave in the stomach both concerning fasted and fed conditions. It is well known that food will effect issues such as gastric emptying and it has been seen for other gelling system that these food effects can lead to substantial delay in the onset of drug action both due to delay in transport from stomach to the small intestinal and due to precipitation of food components that form a film at the tablet surface [37–39].
